# Abstracts From the Infectious Diseases Society for Obstetrics and Gynecology

**DOI:** 10.1155/S1064744996000117

**Published:** 1996


Infectious Diseases in Obstetrics and Gynecology 4:47-57 (1996)
© 1996 Wiley-Liss, Inc.

Abstracts From
the

### Infectious Diseases Society for Obstetrics and Gynecology

### Twenty-Third Annual Meeting
### Hyatt Regency, Beaver Creek, CO
### August 14-17, 1996

### *SESSION 1*

**THE MATERNAL AND FETAL POLYMORPHONUCLEAR CELL CONTRIBUTIONS IN THE PLACENTA AND UMBILICAL CORD OF PATIENTS WITH CLINICAL CHORIOAMNIONITIS AND PRETERM DELIVERY.** M. McNamara, D.O., T Wallis, B.S., S Jacques, M.D., F Qureshi, M.D., B Gonik, M.D. Depts. of Obstetric and Gynecology and Pathology, Hutzel Hosp, Wayne State University, Detroit, MI.
**Objective:** To describe the maternal and fetal polymorphonuclear cell (PMN) contributions in the umbilical cord and placenta in preterm deliveries complicated by clinical chorioamnionitis.
**Study Design:** Five cases of preterm deliveries complicated by clinical chorioamnionitis were retrospectively identified. Histologically severe chorioamnionitis and funisistis were confirmed by hematoxylin and eosin staining. Only male fetuses were studied. Thick sections of tissue were obtained from representative paraffin tissue blocks. Cell suspensions were obtained and cytospin slides were generated. The slides were then prepared for X and Y chromosome probe labeling and fluorescent in-situ hybridization (FISH). One hundred polymorphonuclear cells were identified morphologically and the presence of two XX signals or a single X and Y pair were noted under fluorescent microscopy. The ratio of maternal to fetal PMNs were noted and compared from the placenta and the umbilical cord.
**Results:** Maternal cells accounted for a mean of 86.4% of the total PMNs identified in the placenta. Fetal PMNs accounted for a mean of 99.8% of the PMNs seen in the umbilical cord specimens.
**Conclusion:** In preterm clinical chorioamnionitis, the PMN response observed in the placental mass is predominantly maternal. Conversely, the accompanying funisitis seen on histologic examination is predominantly fetal in origin.

**INTERLEUKIN 1-β, TUMOR NECROSIS FACTOR- α OR LIPOPOLYSACCHARIDE DOES NOT INDUCE APOPTOSIS OF AMNION CELLS IN VITRO.** B.T. Oshiro, R. M. Silver, S.S. Edwin, M. Monga, K. Ward. Depts. of Obstetrics and Gynecology and Human Genetics, University of Utah, Salt Lake City, UT and Dept. of Obstetrics and Gynecology and Reproductive Sciences, UT-HSC, Houston, TX.

**Objective:** Tumor necrosis factor-α (TNF) and other inflammatory mediators are increased in the gestational tissues of women with infection related premature rupture of membranes (PROM) and preterm labor. Also, apoptosis has been reported in fetal membranes taken from pregnancies complicated by PROM and has been proposed as a mechanism of membrane rupture. Since TNF can induce apoptosis in various cell lines, we postulated that intrauterine infection causes apoptosis in amnion cells, leading to PROM. The objective of this study was to determine whether TNF, interleukin 1-β (IL-1), or lipopolysaccharides (LPS) induce apoptosis of amnion cells in culture.

**Study Design:** We obtained fresh placental specimens from patients undergoing elective repeat cesarean sections Amnion cells were isolated and cultured in Ham's F12-Dulbecco's modified Eagle media supplemented with 10% fetal calf serum in 24-well tissue culture plates until confluence. Apoptosis was determined by a DNA fragmentation assay. The cells were incubated with 1 μCi tritiated thymidine for 18 hours, washed and then incubated with medium for 8 hours. Cells were further incubated in quadruplicate in fresh medium with various concentrations of TNF, IL-1, or *E. coli* derived LPS, for 24 hours. The cells were then lysed and the radioactivity determined by liquid scintillation counting. Percent specific fragmentation was calculated expressed relative to control

**Results:** We found no increase in the apoptosis associated with DNA fragmentation in amnion cells treated with IL-1, TNF or LPS

| | IL-1 (% vs Control) | TNF (% vs Control) | LPS (% vs Control) |
|---|---|---|---|
| 0.1 ng/ml | 99.7 | 99.9 | 100 |
| 10 ng/ml | 100 | 99.9 | 100 |
| 100 ng/ml | 99 6 | 99.8 | 99.4 |

**Conclusion:** IL-1, TNF, and LPS do not induce apoptosis in amnion cells in culture at the various concentrations tested. Cytokine-induced apoptosis does not appear to be a mechanism for PROM.

AMNIOTIC FLUID DEFENSIN LEVELS ARE ELEVATED IN PRETERM LABOR PATIENTS WITH SUBCLINICAL INTRA-AMNIOTIC INFECTION. R. Phillip Heine, M.D., Kim Heller, M.D., Leo Mortimer, M.S., Phillip Grieg, M.D.* University of Pittsburgh, Magee-Womens Hospital, Department of Obstetrics, Gynecology and Reproductive Sciences, Pittsburgh, PA. *Greenville Hospital System, Maternal Fetal Medicine Division, Greenville, SC.
OBJECTIVE: Defensins are neutrophil granule proteins released from activated neutrophils in the setting of infection. The purpose of this study was to determine if amniotic fluid defensin levels are elevated in preterm labor patients with subclinical intra-amniotic infection. STUDY DESIGN: Amniotic fluid samples were obtained from 186 pregnant patients with the following clinical characteristics: Group 1 term, no labor (N=50). Group 2 preterm, no labor (N=81). Group 3, preterm labor with intra-amniotic infection (N=20). Group 4, preterm labor without intra-amniotic infection (N=35). Defensin levels were measured by ELISA. Patient groups were compared utilizing the Mann-Whitney U test. RESULTS: Median amniotic fluid defensin levels for the varying patient groups are presented in the table. PatientS with intra-amniotic infection had a significant increase in amniotic fluid defensins when compared to the other groups (p < .0001). An amniotic fluid defensin level of > 400 ng/ml was highly suggestive of infection with a sensitivity of 85% and a specificity of 97%. CONCLUSION: Amniotic fluid defensin levels are elevated in patients with PTL and subclinical intra-amniotic infection. Amniotic fluid defensin levels may be a useful clinical tool for selecting preterm labor patients who might benefit from antimicrobial therapy, closer observation, or early delivery.

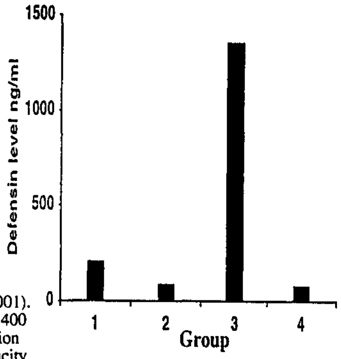

ASSOCIATION BETWEEN EASILY OBSERVED MEMBRANE ROLL CHARACTERISTICS AND HIGH AMNIOTIC FLUID INTERLEUKIN-6 LEVELS AMONG PATIENTS DELIVERING PREMATURELY
DL Patton, JE Hitti, MJ Krohn, SL Hillier, DA Eschenbach, Department of Obstetrics and Gynecology and Medicine, University of Washington, Seattle, and Department of Obstetrics and Gynecology, University of Pittsburgh, Pittsburgh.
INTRODUCTION: Amniotic fluid infection (AFI) and elevated cytokine levels have been associated with prematurity. However, amniocentesis is not routinely performed in the assessment of patients in preterm labor.
OBJECTIVE: To determine whether easily observed characteristics of membrane rolls are associated with increased amniotic fluid (AF) levels of interleukin-6 (IL-6) and AFI among patients delivering at ≤34 weeks.
STUDY DESIGN: The study population consisted of 102 women who presented in preterm labor with intact membranes and delivered at ≤34 weeks gestation. AF was collected by amniocentesis for culture and IL-6 enzyme immunoassay. Formalin fixed, paraffin embedded, membrane rolls were assessed by light microscopy for the presence of any inflammation, chorioamnion sloughing, and necrosis. Logistic regression was used to calculate adjusted OR for the associations between membrane characteristics and an elevated level of IL-6 or a positive culture.
RESULTS: Inflammation, sloughing and necrosis were positively associated with elevated IL-6 levels:

| Membrane Roll Characteristic | IL-6 >2000 pg/ml (n=37) | IL-6 ≤2000 pg/ml (n=65) | Adjusted OR* | 95% CI |
|---|---|---|---|---|
| Inflammation | 33/36 (92%) | 31/63 (49%) | 7.6 | 1.8-29.7 |
| Sloughing | 12/37 (32%) | 14/65 (22%) | 3.2 | 1.1-9.9 |
| Necrosis | 13/37 (35%) | 4/65 (6%) | 5.2 | 1.2-23.2 |

*Adjusted for other factors in tables and gestational age at delivery.
In a similar logistic model, inflammation was also positively associated with AFI (adjusted OR 9.1, 95% CI 1.1-73.7).
CONCLUSION: Easily observed membrane roll characteristics are positively associated with elevated levels of AF IL-6 and AFI. In cases of preterm delivery, light microscopic evaluation of the membrane roll is a simple and inexpensive method for estimating whether AFI has occurred, and may be of prognostic value to the clinician.

BROAD-SPECTRUM BACTERIAL RIBOSOMAL RNA POLYMERASE CHAIN REACTION FOR THE DETECTION OF AMNIOTIC FLUID INFECTION AMONG WOMEN IN PRETERM LABOR. J Hitti, D Riley, MA Krohn, SL Hillier, K Agnew, J Krieger, DA Eschenbach; Departments of Obstetrics/Gynecology and Urology, University of Washington.

OBJECTIVE: To determine the rate of amniotic fluid (AF) infection in preterm labor patients with negative AF cultures, using bacterial ribosomal RNA polymerase chain reaction (rRNA PCR).
STUDY DESIGN: 70 preterm labor patients with intact membranes at 22-34 weeks gestation had AF collected by amniocentesis for culture and interleukin-6 (IL-6) enzyme immunoassay. AF bacterial rRNA PCR for 17 patients with positive cultures were compared with 14 patients with negative cultures and IL-6>2000 pg/ml and 39 patients with negative cultures and low AF IL-6. Kruskal-Wallis ANOVA and paired Mann-Whitney tests with Bonferroni correction were used to examine differences between +culture, -culture/+PCR, and -culture/-PCR groups.
RESULTS: Bacterial rRNA PCR was positive in 15(88%) of 17 patients with positive cultures, 5(36%) of 14 patients with negative cultures and high IL-6, and 1(3%) of 39 patients with negative cultures and low IL-6 ($p<0.00001, \chi^2$). Median (95%CI) cytokine levels, days to delivery and birthweights are summarized:

| | +AF Culture (N=17) | | -Culture/+PCR (N=6) | | -Culture/-PCR (N=47) | |
|---|---|---|---|---|---|---|
| | *Median* | *(95% CI)* | *Median* | *(95% CI)* | *Median* | *(95% CI)* |
| IL-6 (pg/ml) | 25,800 | (5,966-46,400) | 11,958 | (470-49,800) | 190 | (<70-267)* |
| TNF-α (pg/ml) | 876 | (119-1101) | 548 | (<6-7249) | <6 | (<6)* |
| Days to Delivery | 1 | (1-2) | 1.5 | (1-3) | 19 | (13-25)* |
| Birthweight (g) | 900 | (780-1542) | 1679 | (662-2307) | 2695 | (2362-2864)* |

*Kruskal-Wallis ANOVA p<0.0001; paired Mann-Whitney p <0.05 for difference between -culture/ +PCR and -culture/-PCR groups.

CONCLUSIONS: PCR detects AF bacteria in 36% of patients with negative cultures and IL-6 levels > 2000 pg/ml. Cytokine levels and pregnancy outcomes are similar in the +culture and -culture/+PCR groups. The association between AF infection and preterm labor may be underestimated by AF culture.

RESULTS: Twenty-one of 385 women (5.4%) were found to have ASB. Performance of the screening tests are presented in the table below.

| Technique | Sensitivity | Specificity | PPV | NPV |
|---|---|---|---|---|
| ATP | 95 | 50 | 10 | 99 |
| LE | 42 | 77 | 10 | 96 |
| N | 61 | 88 | 23 | 98 |
| LE or N | 76 | 69 | 12 | 98 |

CONCLUSION: ATP bioluminescence is an effective screening test for ASB in pregnancy. Implementation of ATP testing prior to culture, thereby selecting women with the greatest likelihood of ASB, would result in a 50% reduction in urine cultures. Future work will focus on optimizing differential cell lysis in order to improve specificities and further reduce the need for routine culture.

**METRONIDAZOLE TREATMENT OF BACTERIAL VAGINOSIS IN PREGNANCY, AND PRETERM BIRTH: A RANDOMIZED, PLACEBO-CONTROLLED TRIAL.**

HM McDonald[1] PhD, JA O'Loughlin[1] FRACOG, R Vigneswaran[1] FRACP, PJ McDonald[1] FRACP, PT Jolley[1] B Pharm, J Harvey[2] FRACOG, A Bof[3] MBBS, [1]Women's & Children's Hospital, [2]The Queen Elizabeth Hospital, [3]Lyell McEwin Health Service, Adelaide, Australia.

**Objectives** To determine whether metronidazole treatment of bacterial vaginosis(BV) in pregnancy reduces the rate of spontaneous preterm birth (PTB).

**Study Design** Randomised, double-blind, placebo-controlled study of two successive monthly courses of oral metronidazole (400 mg bd for 2 days) in pregnant women at 24 weeks' gestation, with intermediate flora (heavy growth of *G. vaginalis*) or bacterial vaginosis (by Gram stain). Follow-up vaginal swabs were performed at 28, 32 and 36 weeks' gestation. Exclusions included women with age <17 years, multiple pregnancy, or recent antibiotics for vaginal discharge

**Results** 625 women fully completed the study protocol at one of the 4 participating centres. 17(5 5%) of 312 women in the placebo group gave birth preterm (<37 weeks), compared with 16(5 1%) of 313 in the metronidazole group A direct smear indicative of BV was present in 341 women, however the PTB rate was similar in both the placebo (10/165) and metronidazole (10/176) groups. In contrast, treatment with metronidazole in women with obstetric/demographic risk factors for PTB, resulted in a reduction of the PTB rate. Among women with a previous preterm delivery (PPTD), 5(38.5%) of 13 given placebo gave birth preterm, compared with one(7 7%) of 13 given metronidazole Other categories showing reductions in PTB rate were: "single" [10% placebo vs 6.2% metronidazole]· "age < 20 years" [11.4% vs 0%]. and "unemployed" [9.7% vs 3.9%], although these differences were not statistically significant

**Conclusions**: In women at low risk of PTB, treatment of BV in pregnancy appears to result in little or no reduction in the PTB rate, (although the power of this negative conclusion is limited due to sample size). In women with risk factors (especially PPTD), treatment of BV organisms during pregnancy may reduce the PTB rate, however further enrolments are necessary to demonstrate a statistically significant reduction

**ERVICAL ANTIBODIES TO HERPES SIMPLEX VIRUS DURING THE HIRD TRIMESTER OF PREGNANCY.**

KA Boggess MD, DH Watts MD, ZA rown MD, L Corey MD, and RL Ashley PhD University Of Washington ledical Center Depts. of Ob/Gyn & Laboratory Medicine Seattle, WA

BJECTIVE: To examine cervical secretions for anti-HSV IgA and determine elationship to HSV shedding during the third trimester of pregnancy. TUDY DESIGN: HSV-2 seropositive pregnant women had cervical ecretions collected weekly for anti-HSV IgA from 26-31 weeks' until lelivery. Patients collected daily genital specimens for HSV culture and PCR. ESULTS: Of 9 HSV seropositive women (8 HSV-2 only and 1 HSV-1 and ISV-2), 5 (56%) women had a history of symptomatic herpes and 4 (44%) ad no history of genital symptoms. Cervical anti-HSV IgA was detected on 0 (35.7%) of 84 days sampled. Anti-HSV IgA was detected on significantly nore days in women without a history of symptomatic genital herpes (21 of 7, 56.8%) than those with a history of genital HSV (9 of 47, 19.1%) (P<.0001, isher's exact test). No anti-HSV IgA was detected beyond 37 weeks'. Anti-ISV IgA positive days were more likely to be PCR negative (20 of 82, 24%), han PCR positive (8 of 82, 10%) (P=.001, McNemar's test).

| | anti-HSV IgA + | anti-HSV IgA - | TOTAL |
|---|---|---|---|
| PCR + | 8 (10%) | 6 (7%) | 14 (17%) |
| PCR - | 20 (24%) | 48 (59%) | 68 (83%) |
| TOTAL | 28 (34%) | 54 (66%) | 82 (2 without PCR result) |

ONCLUSIONS: HSV-2 seropositive women with no history of ymptomatic genital herpes have anti-HSV IgA present in their cervical ecretions more often than women with a history of symptomatic herpes. he lack of antibody detected beyond 37 weeks' may be secondary to mmunosupression associated with advancing gestation. The possible role f cervical IgA in clearing HSV from the genital tract remains to be etermined.

### SESSION 2

**ATP BIOLUMINESCENCE IS AN EFFECTIVE SCREENING TEST FOR ASYMPTOMATIC BACTERIURIA OF PREGNANCY.**

R. Phillip Heine, M.D., Brad Shumaker, B.S., Robert Moyer, M.D., Harold Wiesenfeld, M.D., C.M. University of Pittsburgh, Magee-Womens Hospital, Department of Obstetrics, Gynecology and Reproductive Sciences, Pittsburgh, PA.

OBJECTIVE: ATP bioluminescence has been shown to be a sensitive marker for symptomatic urinary tract infections. The purpose of this study is to determine if ATP bioluminescence, leukocyte esterase (LE), or nitrate reduction (N) are sensitive screening tests for asymptomatic bacteriuria (ASB) of pregnancy.

STUDY DESIGN: Urine specimens were collected on 385 patients entering prenatal care at Magee-Womens Hospital Outpatient Clinic. Standard cultures were performed using CNA, sheep blood and MacConkey agarose. ASB was defined as a positive culture with > $10^5$ CFU/ml of a significant pathogen. LE and N were tested by dipstick analysis (Chemstrip 2 LN, Boehringer Mannheim). The presence of N or LE greater than trace was considered positive. Bacterial ATP was detected using differential bacterial/mammalian cell lysis followed by the addition of luciferin and luciferace. The resultant light emission was measured in a bioluminometer (New Horizons Diagnostics) with more than 100 relative light units considered a positive screen.

**DO HERPES SIMPLEX VIRUS CULTURES OF THE NEWBORN PREDICT NEONATAL HERPES?**

Brigit V. Brock, MD, Zane A. Brown, MD, Stacy Selke, MA, Lawrence Corey, MD. University of Washington, Seattle, WA 98195.

**Objective:** Culturing mothers for Herpes Simplex virus (HSV) at the time of delivery identifies some, but not all of the infants at risk for developing neonatal herpes. We hypothesized that in addition to maternal cultures, culturing the neonate in the delivery room would enhance our ability to identify infants at risk for neonatal HSV.

**Study Design:** All obstetrical patients without signs or symptoms of herpes had cervical and vulvar cultures obtained on admission in labor. In addition, HSV cultures from the oropharynx of the newborn were obtained in the delivery room. HSV was isolated using diploid fibroblasts by standard technique.

**Results:** Out of 9,667 total deliveries between 1989 and 1993, 6,338 women and 7,285 neonates had cultures obtained. Fifty-seven women had positive cultures, of which 7 had HSV isolated from the cervix, 44 from the vulva, and 6 from both sites. Over 90% of the "missed" maternal cultures were due to women who presented with vaginal bleeding, advanced cervical dilation, imminent delivery, clinical HSV symptoms, or admission for repeat cesarean section. Of the 9,667 deliveries, both maternal and infant cultures were obtained in 5,201. Of these patients, the virologic concordance was:

| | Infant Cultures | |
|---|---|---|
| | **Positive HSV** | **Negative HSV** |
| Maternal Cultures | | |
| Positive HSV | 0 | 47 |
| Negative HSV | 0 | 5154 |

In all of the remaining 2084 infants, the HSV cultures were negative.

During the study period, 4 infants were infected with neonatal HSV. Two of the infants were culture negative and were born to culture positive mothers. The other two infected infants were born to culture negative mothers, but did not receive cultures in the delivery room.
**Conclusion:** The addition of culturing the infant for HSV at the time of delivery did not appear to enhance the sensitivity for detecting infants at risk for developing neonatal HSV.

VAGINAL DEFENSINS AS LESS-INVASIVE MARKERS FOR SEXUALLY TRANSMITTED DISEASES (STD).
Harold C. Wiesenfeld, MD, CM, R. Phillip Heine, MD, Alexander Panyutich PhD, Marijane Krohn, PhD, Richard L. Sweet, MD. Dept. of Obstetrics, Gynecology, and Reproductive Sciences, Magee-Womens Hospital, Pittsburgh, PA, and University of California, Los Angeles.

OBJECTIVES: Less-invasive STD testing strategies using inexpensive diagnostic tests are urgently needed. We evaluated the levels of defensins, neutrophil granule products, from the distal vagina as markers for the presence of STD's.
STUDY DESIGN: Three hundred patients ttending an STD clinic were enrolled. All patients were tested for *N. gonorrhoeae* (GC), *C. trachomatis* (CT), and *T. vaginalis* (TV) by PCR and/or culture. Swabs from the distal vagina were collected, and defensins levels were measured by an ELISA assay.
RESULTS. Overall, 101 women were infected with an STD. Thirty four women were infected with GC, 31 with CT, and 61 with TV. Median vaginal defensins levels are presented in the graph. Levels associated with each organism were significantly greater than those in uninfected women (p<0.001).
CONCLUSION: Defensins are elevated in the distal vagina in women infected with GC, CT, or TV. The use of vaginal defensins as sensitive markers for the presence of STD's may be a less-invasive strategy for STD testing. Expensive diagnostic tests may then be restricted to those women at greatest likelihood of having an STD, as determined by elevated vaginal defensins levels.

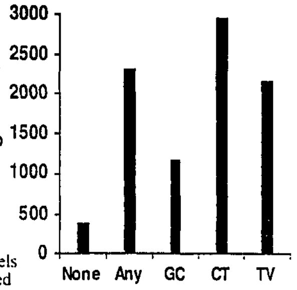

DETECTION OF CHLAMYDIA TRACHOMATIS IN PREGNANT WOMEN BY POLYMERASE CHAIN REACTION (PCR), CERVICAL IgA ANTIBODIES AND ANTIGEN ANALYSIS: RELATION TO PREGNANCY OUTCOME.
SS Witkin, AM Bongiovanni, SR Inglis. Depts. of Ob/Gyn, Cornell University Medical College, New York, NY and Jersey City Medical Center, Jersey City, NJ.

Objectives: To compare the prevalence of cervical C. trachomatis in pregnant women as detected by PCR, IgA antibodies and and antigen detection and ascertain their relation to pregnancy outcome.
Study Design: A total of 211 endocervical samples from 167 women were evaluated. For each sample aliquots were tested for C. trachomatis by PCR (Amplicor, Roche Diagnostics) and for IgA antibodies to C. trachomatis by a 6 minute assay (Chlamydia IgA Rapid SeroTest, Savyon Diagnostics). A third sample was analyzed by the hospital clinical laboratory for C. trachomatis antigen (Chlamydiazyme, Abbott). Pregnancy outcomes were obtained. Laboratory results were not available to the clinician prior to delivery.
Results: C. trachomatis was detected by PCR in 27 (12.8%) samples from 23 (13.8%) women, antichlamydial IgA was present in 37 (17.5%) samples from 32 women (19.2%) while 24 (11.4%) samples from 20 (12.0%) women were positive for chlamydial antigen. Antibody positive, PCR negative samples were retested by a second antibody test and a repeat PCR using samples spiked with C. trachomatis. Compared to PCR, the IgA Rapid SeroTest had a sensitivity of 96.3% and a specificity of 93.5%; the antigen assay had a sensitivity relative to PCR of 74.1% and a specificity of 97.8%. The pregnancy outcomes were 86 (52.1%) patients with term labor (TL), 41 (24.8%) with preterm labor (PTL) and 38 (23.0%) with premature rupture of membranes (PROM). The pregnancies of 11 (47.8%) of the 23 PCR positive women as opposed to 30 (21.1%) of 142 PCR negative women ended in PTL (P=0.009). Only those women positive in the IgA or antigen assays who were also PCR positive had PTL. There was no relation between Chlamydia detection by any assay and PROM.
Conclusions: PCR-detectable C. trachomatis in the endocervix of pregnant women correlates with PTL. In settings where PCR is not available the IgA Rapid SeroTest, which can be performed in 6 minutes without any special equipment, is an accurate and sensitive procedure for detection of cervical C. trachomatis in pregnant women.

### SESSION 3

CHANGES IN VAGINAL FLORA, pH, MYELOPEROXIDASE, AND CERVICAL HIV DETECTION OVER THE MENSTRUAL CYCLE.
DH Watts, MD, SL Hillier, PhD, RC Coombs, MD, S Klebanoff, MD, PhD, MA Krohn, PhD, L Rabe, MS. University of Washington, Seattle, WA; University of Pittsburgh, Pittsburgh, PA.
**Objective:** To evaluate changes in vaginal pH, bacterial flora, and myeloperoxidase levels and cervical HIV detection over the menstrual cycle, in preparation for studies of the effects of vaginal microbicides on the genital tract.
**Study Design:** Women were evaluated twice weekly over the menstrual cycle (total 8 visits) with vaginal pH, Gram stain, quantitative bacterial and genital mycoplasma culture, vaginal myeloperoxidase determinations, and if HIV-positive, plasma and cervical specimens for HIV testing by lymphocyte co-culture and RNA PCR. Subjects did daily basal body temperature charts and had midluteal phase progesterone levels to assess ovulation status.
**Results:** At baseline, of the 8 HIV negative women, 6 had normal flora by Gram stain and 2 were intermediate. Of the 8 HIV positive women, 3 had normal flora, 2 were intermediate, and 3 had bacterial vaginosis. None had GC, Chlamydia, or active HSV at enrollment. Among women with a normal Gram stain at enrollment, *Lactobacillus* concentrations were stable (± 1 log) over the cycle, facultative bacteria including *Enterococcus*, GBS, and *E. coli*, varied over the cycle and anaerobic bacteria were increased during menses. Women with bacterial vaginosis maintained high levels of anaerobic flora throughout the cycle. pH was stable in the absence of bleeding and correlated with Gram stain score. Myeloperoxidase data are incomplete, but levels are variable within each subject and have not correlated with cervical WBC count, pH, Gram stain score or specific flora. Three (43%) of 7 HIV positive women had HIV detected by culture of the cervix at least once, but only 4 (8.5%) of 48 cultures were positive. Four (57%) of 7 had cervical HIV detected by RNA PCR, and 25 (47%) of 53 specimens were positive by PCR. Most women were persistently positive or negative by PCR, but one subject had HIV detected intermittently.
**Conclusions:** Normal variations in vaginal flora and myeloperoxidase levels must be taken into account when assessing the effects of vaginal microbicides. HIV RNA PCR testing appears useful in detecting genital HIV and should be useful for assessing the effect of vaginal microbicides on HIV detection.

**THE MATERNAL-FETAL TRANSFER OF 3TC (LAMIVUDINE) IN THE *EX VIVO* HUMAN PLACENTA.** Keryn M. Dias, M.D., Steven L. Bloom, M.D., Roger E. Bawdon, Ph.D., Dept. Ob/Gyn, Univ. TX Southwestern Med. Ctr., Dallas, TX
**OBJECTIVE:** 3TC (lamivudine) is currently being used as treatment for hepatitis B and in combination therapies for HIV. We sought to study the transfer of 3TC ((-)-2'-deoxy-3'-thiacytidine) across the human placenta both alone and in the presence of AZT (zidovudine).
**STUDY DESIGN:** Nine placentas from term, elective repeat cesarean deliveries were analyzed using the *ex vivo* single cotyledon perfusion system. Antipyrine was utilized as the reference compound for the determination of the clearance indices of 3TC alone and in combination with AZT. 3TC and AZT concentrations in the perfusates and tissues were quantified by reverse phase HPLC.
**RESULTS:** The clearance index of 3TC at a maternal concentration of 1.39 μg/mL was 0.23 ± 0.14. At a peak concentration of 14.6 μg/mL the CI was 0.14 ± 0.06. When the maternal concentration of AZT was 1 μg/mL, the CI of 3TC at the peak and trough concentrations was 0.18 ± 0.08 and 0.19 ± 0.10, respectively. When the concentration of AZT was 10 μg/mL the CI of 3TC was 0.15 ± 0.07 and 0.17 ± 0.09, respectively, for the trough and peak concentrations. In addition, when the perfusion system was closed, there was little accumulation in the fetal perfusate, and in the intervillous space of the placental tissue.
**CONCLUSION:** These data suggest that the CI of 3TC is not altered by the addition of AZT at its peak and trough concentrations. These data also suggest that there is little accumulation of 3TC in the fetal circulation and placental tissue.

**A STUDY OF THE PHARMACOKINETICS OF SINGLE DAILY DOSE GENTAMICIN IN WOMEN WITH POST PARTUM ENDOMETRITIS.**
John A. Sunyecz, MD, Harold C. Wiesenfeld, MD,CM, R. Phillip Heine, MD.
Department of Obstetrics, Gynecology and Reproductive Sciences, Magee-Womens Hospital, 300 Halket Street, Pittsburgh, PA 15213
**Objectives:** Single daily dose therapy has become a popular method of administering aminoglycosides in the non-pregnant patient, due to improved efficacy, decreased toxicity, and lower cost. We investigated the pharmacokinetics of once daily dose gentamicin in women with post partum endomyometritis.

**Study Design:** We studied ten women diagnosed with post partum endomyometritis. All women received 4.5 mg/kg actual body weight of gentamicin intravenously once daily as part of their antibiotic regimen. Serum peak and trough levels were obtained after the first two doses, as well as an eight hour level after the second dose. Serum creatinine levels were monitored, and ototoxicity was assessed clinically.
**Results:** The doses ranged from 255-600 mg (mean=360 mg), and the duration of therapy was 2-5 days. The mean elimination constant (Ke) was 0.105 (SD +/- 0.008), and the volume of distribution (Vd) was 0.4 l/kg (SD +/- 0.07). The mean peak levels after the first and second doses were 11.6 μg/ml (SD +/- 2.3) and 13.0 μg/ml (SD +/- 2.5), respectively. Both trough levels were < 0.3 μg/ml. The mean half-life was 6.6 hrs. There was no nephrotoxicity or ototoxicity observed. Clinical response was seen in 90% of patients; one patient responded with the addition of heparin for presumed septic pelvic thrombophlebitis.
**Conclusions:** Once daily dosing of gentamicin results in pharmacokinetic parameters similar to those observed in non-pregnant patients. No significant drug accumulation or toxicity occured. Therefore, single daily dose gentamicin in the post partum patient appears safe to use at 4.5 mg/kg actual body weight. Further studies are necessary to confirm the safety and efficacy demonstrated in this report.

**COLONIZATION OF RENAL INTERSTITIAL TISSUE WITH *ESCHERICHIA COLI* 075:K5:H-BEARING Dr FIMBRIAE: MUTATION IN THE *dra* OPERON PREVENTED TUBULOINTERSTITIAL NEPHRITIS.** Bogdan Nowicki, M.D., Ph.D.[1,2], Pawel Goluszko, M.D., Ph.D.[1], Steve Moseley, Ph.D.[3], Luan D. Truong, M.D.[4], Anil Kaul, M.D., D.D.S.[1], Stella Nowicki, D.D.S.[1,2] Department of Obstetrics and Gynecology,[1] and Microbiology and Immunology,[2] The University of Texas Medical Branch, Galveston, Texas, Department of Microbiology and Immunology,[3] University of Washington, School of Medicine, Seattle Washington, and Department of Pathology, [4] Renal Pathology Laboratory, Baylor College of Medicine, Houston, Texas.
__Objective:__ *E. coli* that express Dr colonization factors has been recently found in 30% of patients with gestational pyelonephritis. In this study, we investigated the hypothesis that renal interstitial binding mediated by the *E. coli* Dr adhesin *in vitro*, may be important for the development of interstitial colonization resulting in tubulointerstitial nephritis *in vivo*.
__Study Design:__ An insertional *dra* mutant of clinical *E. coli* IH11128 bearing Dr fimbriae was constructed using a suicide vector pGP704. The resulting mutant *E. coli* IH1128 $Dr^-$ lost its attachment capacity to interstitium. The $Dr^+$ parent strain and $Dr^-$ *E. coli* mutant were used to characterize persistence of infection and associated histological lesions in an experimental model of ascending pyelonephritis.
__Results:__ The Dr+ *E. coli* established a long term (52 weeks) colonization of renal tissue. In the $Dr^-$ group, 50% of animals cleared infection within 20 weeks and 100% between 32 to 52 weeks. In the $Dr^+$ group, *E. coli* cells were found to colonize the renal interstitium (detected by hematoxylin-eosin and immuno staining). Fimbrial antigen was detected in the parenchymal regions affected by tubulointerstitial nephritis but not in the non-affected tissue. Histological changes in the $Dr^+$ group included interstitial inflammation, interstitial fibrosis, and tubular atrophy in the kidney tissue. In the Dr group, histological lesions were significantly less severe than those of the $Dr^+$ group and involved a smaller number of animals.
__Conclusions:__ The obtained results are consistent with the hypothesis that mutation within the *dra* region, coding for Dr fimbriae, a factor that mediates binding to renal interstitium, prevents the development of the renal changes characteristically seen in tubulointerstitial nephritis. It remains to be investigated whether the gestational pyelonephritis may contribute to long term complications including hypertension and end stage renal disease. Supported by NIH Grant R01DK42029 (BN).

FETAL MEMBRANE MICROBIAL FLORA AT TERM: NO ASSOCIATION WITH CHORIOAMNIONITIS. Pål Wölner-Hanssen MD, PhD,[1] Eva Morsing MD,[2] Inga Hägerstrand MD, PhD,[3] Elisabeth Holst PhD,[4] Åsa Ljung, MD, PhD,[4] Andreas Herbst MD.[1] Departments of Obstetrics and Gynecology,[1] Pediatrics,[2] Pathology,[3] and Microbiology,[4] University of Lund, Sweden.
**Objectives:** To study relationships between microbial flora of fetal membranes, histopathologic findings of the membranse/placenta/umbilical cord, and clinical symptoms among women delivering at term.
**Study Design**: We studied consecutive women with singleton pregnancy delivering at term during a 3-month period. Immediately after delivery of the placenta, the nurse-midwife separated the fetal membranes. Specimens for culture were collected between the membranes, close to the placental edge with cotton-tipped wooden swabs treated with charcoal. The specimens were kept at room temperature in Stuart's medium and transported to the laboratory within 24 hours (within 48 hours during week-ends). Formalin-fixed membrane roles, and placental and umbilical tissue specimens were studied by a pathologist who was blinded as to clinical and microbiologic findings.
**Results**: We obtained culture specimens from the fetal membranes of 312 (96.9%) of the 330 eligible women who delivered during the study period. At least one bacterial species was recovered in 218 cases (69.9%). Pathogenic microorganisms were recovered from 106 (34%) of the 312 membranes. Culture specimens from the membranes of eight women delivered by elective cesarean section showed no growth. Sixty-two (35.8%) of the first 173 consecutively delivered women had histopathologic evidence of chorioamnionitis. We recovered pathogens from the fetal membranes of 35 (37.2%) women with and 21 (30%) without evidence of chorioamnionitis in the membranes (O.R.= 1.4, p<0.4). Women with fever during labor (n=7) were significantly more likely than those not having fever to have chorioamnionitis (100% vs. 32.1%, O.R.= 16.0).
**Conclusions**: Growth of pathogens between the fetal membranes after vaginal delivery do not predict chorioamnionitis.

**A RANDOMIZED, PROSPECTIVE, THIRD-PARTY BLINDED STUDY OF THE EVALUATION OF AMPICILLIN/SULBACTAM VERSUS AMPICILLIN/ GENTAMICIN IN THE TREATMENT OF CHORIOAMNIONITIS.** **David Soper, MD. Department of Obstetrics and Gynecology, Medical College of Virginia, Richmond, Virginia.**

**Objectives: To assess the efficacy and safety of ampicillin/sulbactam (A/S) compared to ampicillin/gentamicin (A/G) in the treatment of chorioamnionitis.**

**Study design: Patients with chorioamnionitis (temperature > 100°F on two occasions, at least one hour apart or a single temperature greater than 101°F and ruptured membranes and at least one of the following; maternal leukocytosis, maternal tachycardia, fetal tachycardia, foul smelling amniotic fluid) were randomly assigned in double blind fashion to receive either A/S (n = 121) or A/G (n = 120). Clinical failures were defined as those subjects with a persistent temperature elevation (> 100.4°F) for more than 24 hours postpartum. Adverse events in both subject and neonate were assessed.**

**Results: 155 subjects were evaluable for efficacy. 74/79 (94%) of subjects receiving A/S and 73/76 (96%) of subjects receiving A/G were clinically cured (p = NS). Patients undergoing cesarean section (8/53) were more likely to fail therapy than those delivering vaginally (0/102)[p = < 0.001]), more likely to develop postpartum endomyometritis (5/53 vs 0/102 [p = 0.004]), and have a longer hospital duration (5.2 ± 0.7 vs 3.4 ± 0.6 [p < 0.001]). All clinical failures occurred in subjects undergoing cesarean section. Endomyometritis occurred in 3/27 cesarean patients receiving A/S and in 2/26 cesarean patients receiving A/G (p = NS). No serious adverse events due to the study drugs were observed. Minor adverse events were equally distributed between the two study groups. No neonates died from sepsis.**

**Conclusions: A/S and A/G are equally effective and safe in the treatment of chorioamnionitis. Cesarean section patients are more prone to fail to respond to antibiotic therapy due to the development of postpartum endomyometritis.**

PATTERNS OF VAGINAL DOUCHING AND THEIR ASSOCIATION WITH VAGINAL BACTERIOSIS. Bryna Harwood, MD, Robert Mittendorf, MD, Dr PH, Diane Judge, RN, FNP, Sheela Dayal, MD, Cheryl Walker, MD. Department of Obstetrics and Gynecology, University of Chicago, Chicago, IL.

***Objectives:*** We studied the behavior of vaginal douching to determine: 1) the prevalence, methods and rationale for this behavior, and 2) whether douching is associated with an increased prevalence of vaginal bacteriosis. ***Study Design:*** This is a cross-sectional study of women attending outpatient gynecologic clinics at the University of Chicago. Structured interviews were administered by trained female interviewers. Vaginal smears were collected, batched, gram stained, and analyzed by Nugent's criteria for vaginal bacteriosis. ***Results:*** Of the 103 women interviewed, 78.6% had douched in their lifetime, and 74.2% of these women douched at least once a month. Compared with women who did not douche regularly, women who douched at least once a month were more likely to use genital deodorant products (OR=36.6, 95% CI 4.7-286.4, p<0.001), to report the belief that douching can eliminate odors (OR=3.5, 95% CI 1.2-9.9, p=0.015), and to report the belief that douching can prevent vaginal infections (OR=5.3, 95% CI 1.8-16.0, p=0.002). Women who had douched during the past month were more likely to have vaginal bacteriosis than all other women in the study (OR=4.1, 95%CI 1.0, 16.6, p=0.041). ***Conclusions:*** Beliefs about genital hygiene appear likely to underlie most douching: these include deodorizing and preventing infection. We found a significant association between recent douching and vaginal bacteriosis. Any public health efforts designed to discourage douching will have to consider the complex interplay of cultural and racial differences in health beliefs and douching behaviors.

COMPARISON OF CLINICAL MANIFESTATION WITH LAPAROSCOPIC FINDINGS In ACUTE SALPINGITIS

Eschenbach DA, Wolner-Hanssen P, Hawes SE, Pavletic A, Paavonen J, Holmes KK. Department of Obstetrics and Gynecology and Medicine, University of Washington, Seattle

OBJECTIVE: In this report we compared clinical manifestations and tubal abnormalities observed at laparoscopy among women with acute salpingitis.
STUDY DESIGN: We compared the type, and when applicable, the severity of clinical manifestations in 82 women with definite laparoscopic evidence of acute salpingitis. Women with findings other than salpingitis (n=22), normal findings (n=9) or possible, but not definite salpingitis (n=42) were excluded from these analyses.
RESULTS: Two general categories of laparoscopic findings were present: 1) tubal occlusion and moderate to severe adhesions tended to occur together, in 30 patients and 2) pelvic/abdominal exudate tended to occur separately, in 27 patients. Tubal occlusion was positively associated with older age, palpable adnexal mass and negatively associated with rebound tenderness, the tenderness score and the isolation of *Neisseriae gonorrhoeae* and *Chlamydia trachomatis*. Moderate to severe adhesions were positively associated with duration of abdominal pain and negatively associated with the tenderness score. Exudate in the pelvis/abdomen was positively associated with the tenderness score, the white blood count and *N. gonorrhoeae* and negatively associated with the duration of pain, oral contraceptive use and palpable adnexal mass. Reanalyses among patients with no prior history of PID did not change the findings.
CONCLUSION: Clinical manifestation traditionally used to judge the clinical severity of PID partially predict the laparoscopic findings, tend to distinguish those with tubal occlusion and/or moderate to severe adhesion from those with peritonitis, and provide insight into the pathophysiology. However, the predictive value of clinical manifestations are low and not reliable for the individual patient.

TIME TO LIVE BIRTH AFTER PELVIC INFLAMMATORY DISEASE

Lisa A. Lepine, MD, SD Hillis, PhD, PA Marchbanks, PhD, MR Joeseof, MD, PhD, HB Peterson, MD, A Hadgu, Ph.D, L Westrom, MD

OBJECTIVE Our aim was to study the association between severity of pelvic inflammatory disease (PID) at laparoscopy and the length of time to live birth.
STUDY DESIGN: Beginning in 1960, a cohort of 1,730 women in Lund, Sweden who had clinical symptoms of acute PID and who desired pregnancy was followed for up to 24 years All study participants underwent laparoscopy and were assigned to one of four categories normal findings (n = 442), or mild (n = 371), moderate (n = 580), or severe (n = 337) tubal inflammation Cumulative live birth rates were obtained by life-table analysis, and proportional hazards ratios were compared among women with varying degrees of tubal inflammation.
RESULTS. Median time to achieve a live birth according to severity of tubal inflammation at laparoscopic evaluation of PID was 4.0 years for women with no tubal inflammation, 4 3 years for women with mild, 5 6 years for women with moderate, and 11.8 years for women with severe inflammation Three years after the acute episode of PID, 37% with no inflammation, 38% with mild, 26% with moderate, and 16% with severe tubal inflammation were successful in achieving a live birth Cumulative proportion of women achieving a live birth after 12 or more years was 90%, 90%, 82%, and 56% respectively, for those with no, mild, moderate, and severe inflammation After adjustment for age and predisposing factors, women with severe disease and subsequent episodes of PID were eight times more likely to remain childless as those experiencing a single episode with mild tubal inflammation
CONCLUSIONS Increasing severity of PID correlates with longer time in achieving a live birth These data underscore the importance of PID prevention and liberal treatment of suspected PID

### SESSION 4

IDENTIFICATION OF *sac-4* MUTATIONS THAT EFFECT VIRULENCE OF GONOCOCCAL PID STRAINS.

Stella Nowicki, D.D.S.[1,2], Prashanth Ram, M.D.[1], Andrzej Skarpetowski, M.D.[1], Tuan Pham[1,2] and Bogdan Nowicki, M.D., Ph.D.[1,2]. Dept. of Ob/Gyn[1] & Microbiology[2], The University of Texas Medical Branch, Galveston, Texas.

Objectives: Gonococcal PID is a most serious and costly complication of gonorrhea. Pathogenesis of PID is poorly understood. Two major factors contribute to the virulence of gonococci, 1) resistance to the bactericidal effect of normal human serum and 2) attachment to human tissues. C1q-mediated virulence is the first significant difference found between the PID and local strains (Perkin Elmer Award to S. Nowicki's laboratory for the best research in STD in 1995). The *sac-4* DNA region of *N. gonorrhoeae* is known to confer partial serum resistance ($ser^R$). We have recently identified a 344 base pair segment in the 3' end of the *sac-4* region which upon transformation with plasmid pRP350 conferred C1q dependent virulence to laboratory strain F62 . Recently we found two unique local isolates of *N. gonorrhoeae* which carried 344 bp fragment but were avirulent in our pup model. We hypothesize that the 344 bp fragment from local strains carry a point mutation(s) resulting in abolishing C1q dependent virulence.
Study design: *N. gonorrhoeae* strains 1655 and 1653 were used to PCR amplify 344 bp fragments. PCR products were subcloned to appropriate vector. Resultant plasmids pRP1659, pRP1665 were sequenced, and transformed to laboratory strain F62. Virulence of these constructs was examined on pup model.
Results: A 344 bp fragments from local isolates did not confer C1q mediated virulence to *N. gonorrhoeae* F62 upon transformation. In control plasmid pRP350 from PID isolate transformed strain F62 to a C1q dependent virulent strain. Sequencing of plasmids pRP1655 and pRP1659 from local strains and pRP350 from PID strain has been carried out and two deletions and three substitutions were identified. Deletion of the 5' segment of pRP350 that overlap identified mutations abolished C1q dependent virulence.
Conclusions: These results are consistent with the hypothesis that mutations in the 344 bp segment of *sac-4* may effect virulence of gonococcal isolates.

ANTEPARTUM DRUG ABUSE AND PRETERM DELIVERY OF A LOW BIRTH WEIGHT INFANT: IS THE RISK ACCOUNTED FOR BY CO-FACTORS?

Marijane A Krohn, Ph.D., D. Heather Watts, M.D., Sharon L. Hillier, Ph.D., David A. Eschenbach, M.D.

**Objectives**· The purpose of this study was to determine whether the risk of preterm delivery associated with drug abuse during pregnancy could be accounted for by characterstics of drug abusing women such as ethnicity, high number of pregnancies, increased tobacco use, and a high frequency of genital infections.
**Study Design**: Two cohorts of women were compared for their frequency of preterm delivery: (1) 181 women seeking treatment for drug abuse (1990-1994) and (2) 557 non-drug abusing women (1984-1986). Both cohorts had evaluations of genital specimens performed in one laboratory at UWMC. Women participated in personal interviews and had genital specimens obtained between 16 and 30 weeks gestational age. At delivery, a structured medical record review was conducted and preterm delivery was defined as gestational age <37 weeks and birth weight <2500 gm.
**Results**: Drug abusing women were at higher risk of preterm delivery (Unadjusted Odds Ratio (OR) = 3.2, 95% Confidence Interval (CI) = 1.7 - 6.2) Drug abusing women were also more likely to have the following characteristics: older age, non-white race, high number of pregnancies, tobacco use, 60% increase in STD's, and 2.6 fold increase in bacterial vaginosis After multivariable adjustment for competing risks of preterm delivery, drug abusing women were still at increased risk of preterm delivery (OR = 2.6; 95% CI = 1.1 - 6.8) When drug abuse (OR = 4.2; 95% CI 1 5-12) was modelled together with bacterial vaginosis (OR = 3.0; 95% CI 1.0-93), it remained an independent risk for preterm delivery. The risk of preterm delivery among women with both factors was no greater than the separate risks.
**Conclusions**: The risk of preterm delivery associated with drug abuse during pregnancy is independent from characteristics of drug abusing women: bacterial vaginosis, STD's, and demographic and social factors.

PREVALENCE AND CORRELATES OF ANTIBODY TO CHLAMYDIAL HEAT SHOCK PROTEIN IN WOMEN STD CLINIC ATTENDEES AND WOMEN WITH CONFIRMED PID

LO Eckert, SE Hawes, P Wolner-Hanssen, CE Stevens, DM Money, RW Peeling, RC Brunham, KK Holmes, DA Eschenbach, WE Stamm, Department of Obstetrics and Gynecology and Medicine, University of Washington, Seattle, National Laboratory for STD CDC and Department of Medicine, University of Manitoba, Winnipeg

OBJECTIVE: Determine historic, laboratory and laparoscopic factors that correlate with antibody to the 60 kD chlamydial heat shock protein (Chsp-60) in women at risk for chlamydial (CT) cervicitis and acute PID.
STUDY DESIGN: Cross-sectional study of 156 randomly selected STD clinic attendees without clinical evidence of PID and 150 women with PID confirmed by laparoscopy (n=69) or endometrial biopsy (n=81). All women underwent a standard interview, cultures and determinations of antibody to CT using micro-immunoflorescence and of antibody to Chsp-60 using ELISA. A positive Chsp-60 level was an optical density (OD) ≥ 0.2.
RESULTS: Chsp-60 antibody was present in 146 (47%) of the patients. In multivariate analyses, Chsp-60 antibody was related to confirmed PID (OR=2.3, CI 1.3-2.4), age > 20 (OR=2.1, CI 1.1-3.8), CT IgG titers of 1:16-1:128 (OR=2.6, CI 1.2-5.7), CT IgG titers >1:128 (OR=44.7 CI 8.6-233), >10 lifetime sexual parters, non-white race and current OC use, but not to CT culture or IgM titers to CT. Chsp-60 antibody was also related to laparoscopic findings among those with salpingitis confirmed by laparoscopy.

| | Chsp <0.2 (n=30) | Chsp 0.2-1.0 (n=20) | Chsp >1.0 (n=19) | p for trend |
|---|---|---|---|---|
| Occluded tubes | 3% | 50% | 42% | <0.001 |
| Any adhesions | 50% | 75% | 79% | 0.03 |
| Pelvic/abd. pus | 83% | 75% | 53% | 0.02 |

CONCLUSION: Antibody to Chsp-60 appears related to markers of long-standing infection and upper genital tract pathology, but not acute cervicitis.

Effect of Contraceptive Method on Vaginal Flora. SL Hillier, Ph.D.,TM Hooton, M.D., Carol Winter, CRNP, W. Stamm, M.D. University of Pittsburgh, Pittsburgh, PA and University of Washington, Seattle, WA.

**Objective**: To evaluate whether use of oral contraceptives (OC), diaphragms (DI), or cervical caps (CC) alter the vaginal microflora. *In vitro* data has suggested that use of the spermicide nonoxynol 9 could deplete vaginal lactobacilli.

**Study Design**: Women attending a student health clinic who were changing methods of birth control were evaluated prior to starting the new method and for the next four consecutive weeks. Vaginal flora was assessed at each visit, and women recorded frequency of intercourse and use of the birth control method and nonoxynol 9 by diary The group was comprised of predominantly by unmarried Caucasian women, aged 18-30, who were having vaginal intercourse 1-2 times weekly.

**Results**: 213 women (103 OC, 75 DI, 35 CC) were recruited and followed for one month, for a total of 1065 visits Results for DI and CC use were similar, so the groups were combined. Within one week of beginning DI or CC use, vaginal colonization by E. coli increased from 25% (27/111) to 49% (54/110). (P < .05, McNemar's test). The increase in E. coli colonization persisted at one month. Similarly, use of these barrier methods with nonoxynol 9 increased vaginal colonization by Enterococcus from 13% at enrollment to 35% one week after using this new method. However, use of DI and CC did not affect vaginal colonization by lactobacilli (95% vs. 93%), G. vaginalis (36% vs. 40%), anaerobic gram negative rods (40% vs. 55%), Group B streptococci, or yeast. Vaginal colonization by coagulase negative staphylococci decreased following use of barrier methods for one month (57% vs. 37%) There was no effect on the vaginal microflora associated with OC use

**Conclusions**: Use of diaphragms or cervical caps with nonoxynol 9 increases vaginal colonization by E. coli and Enterococcus, but has no adverse effect on vaginal lactobacilli. Use of OC does not alter the vaginal microflora and not increase colonization by yeast.

**AZITHROMYCIN POWDER VERSUS ERYTHROMYCIN IN THE TREATMENT OF CHLAMYDIAL CERVICITIS IN PREGNANCY**

Marcus E. Gunter, M.D., C. David Adair, M.D., J.M. Ernest, M.D., Gayle McElroy, PAC, Dept. of Obstetrics and Gynecology, Bowman Gray School of Medicine, Winston-Salem, NC

**Objectives:** Determine if a single one gram dose of azithromycin (Pfizer Labs, New York, NY) has fewer side effects and better compliance rate than a multiple dose regimen of erythromycin in the treatment of chlamydial cervicitis in pregnancy.

**Study Design:** There is an ongoing randomized clinical trial. All women attending their first and 36 week prenatal visit had cervical swabs taken and analyzed with the Gen-Probe PACE 2 (Gen-Probe, Inc., San Diego, CA) DNA probe for Chlamydia trachomatis. All patients with positive results were asked to participate. Patients were assigned to receive either a one gram oral dose of azithromycin powder or seven days of erythromycin base, 500 mg q.i.d. Patients unable to tolerate original randomization medications due to gastrointestinal side effects were allowed to crossover to the opposite study medication. Test of cure swabs were obtained two weeks after completion of treatment. Patients testing positive for Chlamydia trachomatis at follow-up were considered treatment failures and retreated with the other agent. The incidence of side effects and compliance rates were assessed by patient questionnaire at the two week follow-up visit.

**Results:** Forty-seven women have been randomized.

| Variable | Azithromycin N=22 | Erythromycin N=18 | p* |
|---|---|---|---|
| Compliance | 22 (100%) | 8 (44.5%) | ≤0.001 |
| Gastrointestinal side effects | 3 (14.3%) | 11 (61.1%) | ≤0.001 |
| Positive follow-up cultures | 0 (0%) | 1 (5.3%) | NS |
| *= $\chi^2$; data expressed N (%) | | | |

Twenty-seven percent of patients treated with erythromycin were unable to tolerate the medication due to gastrointestinal side effects and required crossover to azithromycin. No patient in the azithromycin group required crossover.

**Conclusions:** Azithromycin powder is better tolerated and has a higher compliance rate than erythromycin in the treatment of chlamydial cervicitis in pregnancy.

**A POPULATION BASED STUDY OF SEXUAL ACTIVITY AMONG 3600 PREGNANT WOMEN AND ASSOCIATIONS WITH MICROBIOLOGICAL FINDINGS IN THE GENITAL TRACT.**

P. Thorsen, N. Ebbesen, I. P. Jensen, M. Arpi, A. Bremmelgaard, B. Jeune, J. G. Westergaard & B. R. Møller. Dept. of Ob/Gyn, Odense University Hospital, Denmark.

**OBJECTIVE:** To evaluate of sexual activity during pregnancy and possible influences of sexual activity on the microbiology of the genital tract.

**STUDY DESIGN:** Three thousand six hundred pregnant women, attending the Department of Obstetrics, Odense University Hospital, Denmark, were asked to participate in the study. The study included a pelvic examination and samples obtained for *Chlamydia trachomatis*, genital mycoplasmas, *Trichomonas vaginalis* and aerobe and anaerobic bacteria at enrolment. Further subjects were asked to fill out three questionnaires; at enrolment, at the 30th week of gestation and at birth. Women who did not complete testing were excluded. Sexual activity was monitored by identical detailed questions in each questionnaire. A selected group of pregnant women (n=539) were asked to go through additional pelvic examinations during 2nd and 3rd trimester and at birth.

**RESULTS:** Two thousand and nine hundred and twenty seven (81.3%) completed the study. Mean gestational age at enrolment was 17 1/7 weeks. The group with high initial sexual activity (more than 2 sexual intercourses per week during the prior 4 weeks) decreased gradually from a proportion of 22.9% at inclusion to 7.6% at birth. Test microorganisms were found more frequently in the group with high sexual activity: *Chlamydia trachomatis* (3.4% vs 1.8%; crude OR 1.9, 95% CI 1.1 - 3.2), *Mycoplasma hominis* (10.6% vs 4.1%; crude OR 2.0, 95% CI 1.5 - 2.7), *Gardnerella vaginalis* (44.7% vs 32.6%; crude OR 1.4, 95% CI 1.2 - 1.7) and *Bacteriodes* spp (7.5% vs 4.5%; crude OR 1.7, 95% CI 1.2 - 2.4). Similarly, women with more than 3 test microorganisms (except *Lactobacillus* spp.) isolated from the genital tract at inclusion, during 2nd and 3rd trimester and at birth were more likely to be among the highly sexually active participants in comparison to the remaining participants (p < 0.05).

**CONCLUSIONS:** 1) Sexual activity decreases 3-fold during pregnancy among women with the most frequent coitus. 2) There is a strong association between higher sexual activity and prevalences of various microorganisms isolated from the genital tract and (3) the number of test microorganisms isolated.

The type and frequency of intercourse and the number of partners during pregnancy likely play primary and secondary roles in reproductive tract microecology and risks of infection associated pregnancy and morbidity.

### SESSION 5

PREVENTION EARLY-ONSET GBS SEPSIS: IMPLEMENTATION OF THE PROPOSED CDC GUIDELINES

Richard L. Sweet, M.D., Beverly Brozanski, M.D., Faith DiBiasi, B.S., M(ASCP), Judith Jones B.S.N.

**Objectives**: The purpose of the study was to document the impact of the introduction into clinical practice of the proposed CDC guidelines for prevention of early onset GBS disease.

**Study Design**: A retrospective study comparing the prevalence of early onset GBS sepsis (positive blood culture GBS) at Magee-Womens Hospital prior to the institution of CDC guidelines (January 1, 1992-June 30, 1995) with the prevalence of GBS sepsis following introduction of the CDC protocol (October 1, 1995-March 31, 1996). The microbiology laboratory data base was reviewed for all positive GBS blood cultures from the nursery, as were the medical records of blood culture GBS positive newborns.

**Results**: From January 1, 1992-June 30, 1995 there were 36 cases of early onset GBS sepsis among 31,133 births for a rate of 1.15 per 1000 live births. After introduction of the CDC protocol there was 1 case of early onset GBS sepsis in 5500 births or .18 per 1000 live births (p=0.03). During these time periods the prevalence of maternal GBS vaginal colonization remained the same (27% vs 25%). By March, 1996 intrapartum prophylaxis for GBS was the most common new antibiotic order accounting for 36% of new antibiotic orders at MWH. Post introduction of the CDC

protocol, IV penicillin made up 83.2% of these orders, clindamycin 13.2%, erythromycin 0.5%, amyicillin 1.3% and other 1.8%.
**Conclusions**: The proposed CDC guidelines for prevention of early onset GBS infection in newborns is very effective, reducing early onset GBS > 5-fold. Institution of the CDC protocol was easily accomplished in a large community hospital where two-thirds of deliveries are by private attendings.

### DOES THE USE OF AMPICILLIN VERSUS PENICILLIN FOR THE PREVENTION OF NEONATAL GROUP B STREPTOCOCCAL (GBS) INFECTION RESULT IN HIGHER RATES OF COLONIZATION WITH RESISTANT ORGANISMS?

Belady P, Graham S, Lezotte D, Merenstein G, Gibbs R University of Colorado, Denver, CO.

**Objectives:** Both ampicillin and penicillin G are recommended for GBS prophylaxis but ampicillin may exert selection pressure for resistant organisms. We hypothesize that neonates of pregnant women treated with intrapartum ampicillin will have significantly higher colonization rates with aerobic gram negative rods (AGNR) compared to neonates of mothers receiving penicillin G.
**Study Design:** A prospective, randomized, unmasked trial of intrapartum chemoprophylaxis was undertaken. Included were women at risk for delivering an infant with GBS infection per ACOG guidelines. Women were randomized by month of admission to receive ampicillin (2g IV followed by 1g every 4 hrs) or penicillin G (5 million u IV every 4 hrs). Prior to initiation of antibiotic treatment, cultures were obtained from the rectum and distal vagina and placed in selective broth media and Culturette tubes Cultures were similarly obtained from the throat, ear and anus of the neonates. GBS, S aureus, enterococcus, and AGNR were then isolated using standard microbiologic techniques. Data were analyzed using Chi-Square and Cochran-Mantel-Haenszel tests.
**Results:** The treatment groups were not significantly different with respect to maternal age and race, gestational age and the use of other antibiotics during pregnancy. Overall, there was no significant difference in neonatal rates of colonization with 1. AGNR (Amp 19/108 vs PCN 28/109), 2. GBS (Amp 1/108 vs PCN 2/109), 3. Enterococcus (Amp 10/108 vs PCN 10/109) and 4. S. aureus (Amp 3/108 vs PCN 4/109) In a subgroup analysis of mothers and neonates with paired cultures, the following differences in rates of neonatal colonization were noted between the two antibiotic groups:

| Antibiotic | Maternal | | | Neonatal | | |
|---|---|---|---|---|---|---|
| | +AGNR | Amp Res | +GBS | +AGNR | Amp Res | +GBS |
| Ampicillin (n=31) | 18 (58%) | 9 (29%) | 5 (16%) | 2 (11%) | 2 (22%) | 0 (0%) |
| Penicillin G (n=29) | 25 (86%) | 13 (45%) | 3 (10%) | 10 (40%) | 5 (38%) | 1 (33%) |
| Relative Risk (vertical transmission with PCN) | | | | 2.6 (1 0-7 4) | 2.0 (0 6-6 4) | 4 5 (0 2-85 1) |

**Conclusions:** 1) There was a higher than expected rate of maternal colonization with -AGNR 2) Ampicillin did not result in selection pressure towards AGNR, ampicillin-resistant AGNR or other organisms. 3) There was a trend toward increased transmission of AGNR with the use of penicillin We speculate that the selection of resistant organisms may occur prior to the use of intrapartum chemoprophylaxis and that intrapartum ampicillin may decrease vertical transmission of AGNR.

### MATERNAL GROUP B STREPTOCOCCAL (GBS) CULTURE STATUS OF INFANTS GBS POSITIVE AT DELIVERY

Beverly Brozanski, M.D., Marijane A. Krohn, Ph.D., Faith DiBiasi, B.S., M(ASCP), Judith Jones B.S.N., R.N.C., Sharon L. Hillier, Ph.D., Richard L. Sweet, M.D.

**Objectives**: The purpose of the study was to evaluate maternal determinants of infant Group B Streptococci colonization at delivery.
**Study Design**: A cohort of 536 mother/infant pairs were enrolled at Magee-Womens Hospital from July 1995 to February 1996. Infants had specimens taken before 12 hr of life for the isolation of GBS from the outer ear, throat, anus, and umbilicus. Specimens were grown on solid media following broth enrichment. Medical records were reviewed to determine the mother's antepartum (AP) GBS culture and intrapartum (IP) chemoprophylaxis status. A subgroup of 216 mothers also had IP vaginal specimens taken for GBS isolation when they were admitted for delivery.
**Results**: Maternal GBS colonization rates in the AP and IP were 26% and 24%, respectively. Seven percent (38/536) of infants were GBS positive from at least one site. Infants were more likely to be positive if maternal AP cultures had been positive (12% versus 5%; P=0.02) but 56% of positive infants were born to culture negative mothers. Infants treated with IP antimicrobials for $\geq$ 4 hr had a 54% reduction in colonization (95% CI 0.2-1.3) compared to infants whose mothers were untreated or treated <4 hr AP cultures were 77% sensitive and 82% specific for predicting women positive at delivery but 23% of women who were positive at delivery were negative earlier in pregnancy. Women who were positive at any testing time were more likely to have a culture positive infant when compared to those who were negative at both testing times (19% versus 0.7%; P < 0.0001)
**Conclusions**: Antepartum maternal specimens taken for the isolation of GBS are inadequate predictors of which women will be positive at delivery. Negative antepartum maternal culture status may result in false reassurance that the infant is not at risk for invasive GBS disease.

### SECRETORY LEUKOCYTE PROTEASE INHIBITOR (SLPI) IN VAGINAL SECRETIONS IS DEGRADED BY CYSTEINE PROTEASES OF *TRICHOMONAS VAGINALIS*.

Deborah Draper, Ph.D., William Donohoe, B.S., R. Phillip Heine, M.D. Magee-Womens Research Institute and University of Pittsburgh, Pittsburgh, PA.

The mechanism of enhancement of acquisition of HIV infection by co-infection with sexually transmitted diseases (STDs) is unknown. SLPI, a serine protease inhibitor, is known to protect oral mucosal surfaces from transmission of the AIDS virus through its inhibition of the protease activity required for HIV infection of mucosal monocytes and macrophages. However, little is known about SLPI function in the genital tract. **Objective:** This study was undertaken to determine if the abundant, secreted cysteine proteases of *T. vaginalis* degrade purified SLPI or SLPI found in vaginal secretions and render it non-functional. **Study Design:** Proteases were induced from 3 isolates of *T. vaginalis* collected from pregnant patients. Protozoan-free, supernatant proteases were fractionated by isoelectric point over a pH gradient of 3.0 to 9.0 using a BioRad RotopHer. Dialyzed, pH gradient fractions of proteases were mixed with human recombinant SLPI (2 ug) or with freshly collected vaginal secretions from normal women (n=3) and incubated at 37°C for 20 hours. The degradation patterns of the mixtures were followed by SDS-PAGE and Western immunoblot analysis using SLPI specific polyclonal antiserum. Protease inhibitory function of SLPI was tested using serine protease peptide-specific substrates in the presence or absence of Trichomonas proteases and excess E-64 inhibitor. **Results:** Two *T. vaginalis* proteases ($M_r$ of 32 and 100kDa) with isoelectric points of approximately pH 7.0, completely degraded the purified SLPI Western signal within 20 hours. These proteases were also able to degrade endogenous SLPI in vaginal secretions. Cysteine protease inhibitor, E-64, completely protected recombinant SLPI from degradation or loss of functional activity. **Conclusions:** These studies demonstrate that *T. vaginalis* cysteine proteases can degrade SLPI in vitro, and could play a role in the *in vivo* degradation of SLPI, thereby rendering the vaginal immune cells more susceptible to HIV infection. These findings could explain the increased risk of HIV acquisition seen in association with *T. vaginalis* infection and could provide a mechanism for enhancement of HIV infection by other non-ulcerative STDs.

### THE EFFECT OF *CHLAMYDIA TRACHOMATIS* AND INFLAMMATORY LEUKOCYTES ON HIV-1 REPLICATION IN VITRO

Daniel V. Landers, MD, John P. Mills, Jr., BS, MT(ASCP), Faith DiBiasi, BS, M(ASCP), Julius Schachter, Ph.D[r].

University of Pittsburgh and Magee-Womens Research Institute and University of California, San Francisco[r].

**Objectives:** Epidemiological studies have shown that *Chlamydia trachomatis* (CT) infection is a risk factor for HIV acquisition. Polymorphonuclear leukocytes (PMNs) and mononuclear cells (PBMCs) are found in the female genital tract in response to CT infection. We studied the effects of CT extracts and inflammatory leukocytes on HIV-1 replication in vitro.
**Study Design:** Chronically HIV-1 infected monocytic cells (U1) were incubated with CT (human E strain) extract, PMNs, or PBMCs. HIV replication was determined by p24 antigen measurement at 24, 48, 72, and 96 hours.
**Results:** After 96 hours of incubation HIV replication was increased 25 fold with PBMC coincubation (p<0.01), 48 fold with PMN coincubation (p<0.01), 27 fold with CT extract and PBMC coincubation (p<0.01), and 33 fold with CT extract and PMN coincubation. Table 1 shows data for 24, 48, 72, and 96 hours:

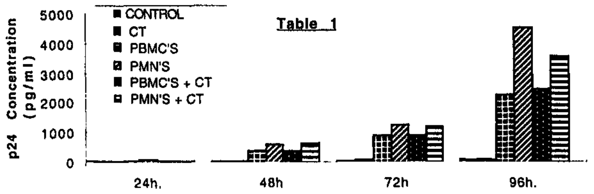

**Conclusion:** Inflammatory cells found in the female genital tract, in association with CT infection, significantly enhance HIV replication in vitro. CT alone or in combination with inflammatory cells did not further enhance HIV replication. This data suggests that the inflammatory response to CT infection is in part responsible for increased risk of HIV acquisition. Further studies are needed to elucidate this complex interaction.

### VAGINAL INFECTIONS IN HIV

Helfgott, A., M.D, Eriksen, N., M.D., Myers, G., CNM, Lorimor, R., Ph.D., Blanco, J., M.D.

**Objectives:** In some reports, HIV-infected women have an increased incidence of recurrent vaginal infections. The purpose of this retrospective review was to identify the rate of vaginal infection and treatment response in a cohort of HIV-infected women in Houston, Texas.

**Study Design**: We reviewed medical records of 96 HIV-infected women who had 149 visits from July 1994 to March 1996. We recorded rate and type of vaginal infections, the therapeutic regimens and immune status of all patients.

**Results**: The mean (± S.D.) age for the entire group was 26.3 ± 7.7 years, and the ethnic composition was African-American 81.3%, Hispanic 10.4% and Caucasian 8.3%. There were 52 patients with 59 episodes of vaginal infection.We found bacterial vaginosis in 35, Candidiasis in 15 and Trichomonis in 9. The overall failure rate was 3 of 59 (5.1%). The failures all occurred in patients who received oral therapy (2 with B.V. and 1 with Candida). One therapeutic failure had a CD4 count of > 500, the other two had CD4 counts < 200. In this study there was no association between the occurrence of vaginal infections and immune status (NS). In addition, there were no associations between CD4 counts and treatment failure (NS).

**Conclusion**: This preliminary review demonstrates a 5.1% treatment failure rate for vaginal infections in HIV-positive women. We found no significant association between treatment failures, therapeutic regimens or immune status.

### SESSION 6

### TNF-A AND IL-6 PRODUCTION IN THE CHORIOAMNION AT TERM BY MACROPHAGES OF MATERNAL AND FETAL ORIGIN AS DETERMINED *IN VIVO* BY mRNA *IN SITU* HYBRIDIZATION AND IMMUNOHISTOLOGY

T Roos, MD,[1] P Myerson, MD,[3] SL Hillier, PhD,[2] G Cadd, PhD,[2] A Gown, MD,[4] G Firestein, MD,[5] DA Eschenbach, MD[2]

Dept of OB/GYN, University of Wuerzburg, FRG[1] and University of Washington, Seattle, WA,[2] Dept of Pathology, Fred Hutchinson Cancer Research Center,[3] and University of Washington, Seattle, WA,[4] and Dept of Medicine, University of California, San Diego, CA[5]

OBJECTIVES: The origin of macrophages associated elevated amniotic fluid (AF) cytokine (CK) levels of tumor necrosis factor-alpha (TNF-a) and interleukin-6 (IL-6) was examined in term pregnancies.
STUDY DESIGN: 33 women undergoing cesarean section had specimens of AF, chorioamnion (CA), decidua (D) and placenta (Plac) collected at delivery. AF, CA and Plac were cultured. AF TNF-a and IL-6 concentrations were determined by ELISA. Immunohistologic differentiation was performed on all tissues for T- and B-lymphocytes and macrophages using antibody markers and/or granulocytes (PMN) identified by morphology. TNF-a and IL-6 producing cells were identified by mRNA *in situ* hybridization using $^{35}$S labeled probes. Fetal macrophages were identified by combined *in situ* immunohistology and hybridization using macrophage specific antibody HAM56 and a DNA probe for Y-chromatin.
RESULTS: Both AF infection and labor were associated with elevated AF levels of TNF-a and IL-6. Macrophages and PMN's were the predominant cell types in the CA. IL-6 mRNA signal in the decidua was related to labor while IL-6 mRNA signal within macrophages in the amnion and chorion was related to a positive AF and CA culture. Macrophages in the amnion were related to positive AF culture. Using the double stain technique, Y-chromatin positive macrophages were present in all 19 pregnancies with a male fetus and in none of 14 pregnancies with a female fetus (p<0.001).
CONCLUSIONS: Fetal amnion and chorion macrophages synthesize CK in response to infection. Most of these macrophages in the amnion and chorion appear to be of fetal origin.

### DETECTION OF *TOXOPLASMA GONDII* IN AMNIOTIC FLUID BY A POLYMERASE CHAIN REACTION/ENZYME-LINKED IMMUNOSORBENT ASSAY (PCR/ELISA).

J Jeremias, WJ Ledger, and SS Witkin, Dept. of Ob/Gyn, Cornell University Medical College, New York, NY

Objectives: Maternal exposure to *Toxoplasma gondii* during pregnancy may result in congenital infection and serious sequela in the neonatal period or years after birth. However, there is little consensus about screening during pregnancy, and the tests used to establish a fetal diagnosis of toxoplasmosis are complex and time consuming. Prenatal diagnosis of congenital toxoplasmosis is based on ultrasonography, fetal blood sampling or inoculation of cultured cells or mice with amniotic fluid. Given the risk associated with fetal-blood sampling and the delay in obtaining definitive results with conventional parasitologic methods or ultrasound, better methods are needed. *T. gondii* has been diagnosed by PCR but detection of the amplified product by an ELISA has, to our knowledge, not been reported. ELISA detection has increased sensitivity over other methods.
Study Design: Using human amniotic fluids that had been spiked with *T. gondii*, we established parameters for the amplification and detection of a fragment of the 35-fold repetitive B1 gene.The 115-base pair product, digoxigenin-labeled, was hybridized to a biotinylated internal probe and detected by ELISA. The hybridization validated the specificity of the amplified PCR product. The possible presence of PCR inhibitors in individual amniotic fluids was evaluated by amplifying a portion of the human beta actin gene.
Results: *T. gondii* DNA was readily detected in amniotic fluids by our PCR/ELISA. PCR analysis of DNA extracted from various bacteria, yeast, and viruses using the *T. gondii* primer pairs did not result in amplification of any crossreacting DNA. Similarly, amniotic fluids obtained for genetic analysis were uniformly negative for *T. gondii*.
Conclusions: The PCR/ELISA will allow for the definitive diagnosis of congenital *T. gondii* infection in one day. This will reduce the incidence of unnecessary pregnancy terminations based on uncertainty of fetal infection as well as allow the prompt initiation of antibiotic treatment to infected fetuses.

### RISK OF HEPATITIS B TRANSMISSION AFTER AMNIOCENTESIS IN CHRONIC HEPATITIS B CARRIERS.

James Alexander, M.D., Ronald Ramus, M.D., Greg Jackson, M.D., Barbara Sereceley, R.N., George Wendel, M.D. The University of Texas Southwestern Medical Center at Dallas, Dallas, Texas

**Objectives**
The risk of transmission of hepatitis B virus (HBV) from amniocentesis in HBV carriers has not been thoroughly investigated. In one report from Taiwan, the risk of perinatal HBV transmission in HBV carriers was not higher after amniocentesis compared to a control group of HBV carriers. Immunoprophylaxis failures occured in 10% of infants in both groups. Our objective was to measure the risk of transmission of HBV in chronic carriers who undergo amniocenteses at our hospital.

**Study Design**
This study was a prospective, longitudinal study, and data were collected about women who were HBV carriers and underwent amniocentesis. The infants of these women were followed in a special clinic from birth to one year of age. Maternal data examined included HBV antigen and antibody status, liver function tests (LFTs), and the amniocentesis procedure report. Pediatric data was obtained from clinic records including the neonatal and 12 month HBsAg and vaccine record.

**Results**

25 women were identified. Two of 25 neonates were stillborn unrelated to hepatitis, and 5 infants were lost to follow-up, leaving 18 mother-child pairs to evaluate. All 18 women were chronic HBV carriers at the time of amniocentesis and delivery. No mother had abnormal LFTs, and only 1 of 18 women was positive for HBeAg. 10 amniocentesis were for advanced maternal age, and 5 were for abnormal MSAFP screening. None of the amniocenteses were recorded as bloody, and the placenta was anterior in 5 of 18 procedures. None of the 18 infants (95% CI:0-18.5%) were positive for HbsAg during the first month of life or at 12 months of age. All infants received HBV vaccine and HBIg immunoprophylaxis.

**Conclusion**
The risk of transmission of HBV to the fetus after amniocentesis in women who are HBV carriers is low. Immunoprophylaxis in these infants was successful.

### AMNIOTIC FLUID CONCENTRATIONS OF TNF-α VERSUS TGF-$\beta_2$ IN A RABBIT MODEL OF INFECTION-INDUCED PRETERM BIRTH.

K. K. Leslie, M.D.[1] L. Reznikov, M.D., Ph.D., [1] R. McDuffie, M.D.[2] F. Cominelli, M.D.[3] L. Melani, Ph.D.[3] R. S. Gibbs, M.D.[1] Department of Obstetrics and Gynecology, the University of Colorado Health Sciences Center[1] and Kaiser Permanente,[2] Denver, CO; the Department of Gastroenterology, the University of Virginia School of Medicine, Charlottesville, VA.[3]

Objectives: In the rabbit model of infection-induced preterm labor, as well as in women with labor associated with inflammation and infection, the expression of pro-inflammatory cytokines such as tumor necrosis factor-alpha (TNF-α) and interleukin-1 (IL-1) stimulate prostaglandin synthesis leading to uterine contractions. On the other hand, endogenous anti-inflammatory proteins such as transforming growth factor-beta$_2$ (TGF-$\beta_2$) and interleukin-1 receptor antagonist (IL-1ra) inhibit prostaglandin production. We hypothesize that uterine contractility depends upon a delicate balance between stimulatory and inhibitory cytokine-like factors in the uterine environment; the purpose of this study was to compare the concentration of TNF-α to the anti-inflammatory factor TGF-$\beta_2$ in amniotic fluid using the rabbit model. Study Design: New Zealand white rabbits at approximately 70% gestation underwent hysteroscopic inoculation of E. coli and were sacrificed at varying times after bacterial introduction. Amniotic fluid was collected and assayed for protein levels of TNF-α and TGF-$\beta_2$ by enzyme-linked immunoassay (ELISA, R&D Systems, Minneapolis, MN). Results: TNF-α rose from 0.44 pg/ml at time 0 to 2.5 pg/ml by 16 hrs ($P \leq 0.019$). TGF-$\beta_2$ was significantly higher than TNF-α at time 0 (610 pg/ml versus 0.44 pg/ml, $P \leq 0.01$), and did not change over time. Conclusions: (1) TNF-α concentration, as measured by ELISA, rises significantly with infection. (2) Compared to TNF-α, TGF-$\beta_2$ is present at high levels in the amniotic fluid but does not change with infection. We speculate that rising TNF-α in the face of unchanging TGF-$\beta_2$ creates an imbalance between pro- and anti-inflammatory cytokines which leads to uterine contractions.

SEROLOGICAL RESPONSE TO THE CHLAMYDIAL 60 kDa HEAT SHOCK PROTEIN IN WOMEN WITH TUBAL FACTOR INFERTILITY by K Ault, B Statland, M Smith King, D Dozier and J Gunter. University of Kansas Medical Center Kansas City, KS USA

Objective: to study the relationship between serological response to chlamydial 60 kDa heat shock protein (hsp 60) and tubal factor infertility (TFI).

Study Design: Twenty three women with TFI and 33 women with male factor infertility (controls) were studied. TFI was defined as unprotected intercourse for one year with hydrosalpinx or distal tubal occlusion on hysterosalpingogram or at laparoscopy. Demographic data was gathered by chart review. Patients' sera were tested for antibodies to the hsp 60 using an enzyme linked immunoabsorbent assay (ELISA). Stepwise logistic regression was performed on patient's age, race/ethnicity, self reported history of *Chlamydia* or pelvic inflammatory disease, history of ectopic pregnancy and status of anti-hsp antibodies with the hsp 60 ELISA.

Results: Eighteen of the 23 women with TFI had a positive result on the hsp ELISA (78.6%). Significant risk factors for TFI were age > 35, (odds ratio = 2.23, p = 0.0086), "non-white" race (OR = 2.05, p = 0.0001), history of ectopic pregnancy (OR = 2.70, p = 0.0008) and a positive hsp 60 ELISA (OR = 3.26, p < 0.0001).

Conclusions: A positive serological response to the hsp 60 is strongly correlated to TFI. The potential value of the hsp ELISA test should be evaluated in a prospective study of infertile women.

DISTRIBUTION OF ANTIBODIES TO CHLAMYDIA TRACHOMATIS IN FOLLICULAR FLUID AND SERA OF WOMEN UNDERGOING IN VITRO FERTILIZATION (IVF). A. Neuer, K.-N. Lam and L. Kiesel. Dept of Ob/Gyn, University of Tuebingen Women's Hospital, Tuebingen, Germany

Objectives: Unsuspected C. trachomatis infection has been associated with adverse IVF outcome. The aim of this study was to determine the prevalence and distribution of antichlamydial antibodies in follicular fluid and sera of asymptomatic women undergoing IVF.
Study Design: Paired sera and follicular fluids (FF) were obtained at the time of oocyte aspiration from 149 women undergoing IVF. FF were free of visible blood contamination. Samples were assayed for IgG and IgA antibodies to C. trachomatis by two different ELISA assays employing either a recombinant Chlamydia-specific lipopolysaccharide (LPS) fragment or the chlamydial major outer membrane protein (MOMP). All subjects were tested for cervical C. trachomatis by DNA probe; FF were tested for C. trachomatis by polymerase chain reaction (PCR) and ligase chain reaction (LCR).
Results: Sera from 90 (60%) subjects were positive for antichlamydial IgG utilizing the LPS assay; 54 (36%) were positive for antichlamydial IgG in the MOMP assay. Similarly, LPS-directed and MOMP-directed IgA antibodies in sera were detected in 50 (34%) and 21 (14%) of the subjects, respectively In FF, IgG antibodies to MOMP were also more prevalent than IgA antiMOMP antibodies (46% vs. 9%). However, unlike in sera IgA antiLPS antibodies were present in FF from 107 (72%) women as opposed to only 50 (34%) being IgG antiLPS positive (P<.0001). In 54 women antichlamydial IgA was detectable in FF but not in the corresponding serum. All cervical and FF samples were negative for C. trachomatis by PCR and LCR.
Conclusions: Similarities in the prevalence of IgG and IgA antibodies to MOMP in sera and FF suggest that these antibodies enter the FF primarily by transudation from the circulation. The greatly increased prevalence of IgA antibodies to chlamydial LPS in FF suggests that a localized humoral immune immune response to this antigen may be induced in the upper genital tract in the absence of a systemic immune response. Perhaps differential expression of chlamydial antigens during a chronic upper genital tract infection vs. an acute lower tract infection could account for the variability in antibody specificity.

**PROPHYLACTIC ANTIBIOTICS AND ENDOMETRIAL MICROFLORA.** Edward R. Newton, M.D.,* Dept. Ob/Gyn/Reprod Sciences Univ Texas Med Schl at Houston, TX.
**OBJECTIVE:** To determine the effect of prophylactic antibiotics on the endometrial microflora at the diagnosis of post-cesarean endometritis.
**STUDY DESIGN:** The records of 691 subjects involved in comparative trials of therapeutic antibiotics for post-partum endometritis between 1989-1994 were reviewed. Intravenous prophylactic antibiotics were ampicillin (AMP) 2 g q6h 1-3 doses, cefazolin (CEF) 2 g q6h 1-3 doses or other (cefotetan 2 g 1 dose, Unasyn® 3 g q6h x 1-3 doses). Endometritis was diagnosed with a post-partum temperature ≥ 38°C recorded twice, 6 hours apart and associated with localizing signs and symptoms. Endometrial cultures were obtained by a sheathed, injection/aspiration technique. Aerobes and anaerobes were isolated by standard microbiologic techniques.
**RESULTS:** Culture results were available on 682 patients. 467/691 (68%) had a cesarean section. 153 received AMP prophylaxis, 101 received CEF, 17 received other and 196 received no prophylaxis. (Table 1; *P<0.01 VS None)

| ENDO ISOLATE | AMP | CEF | OTHER | NONE |
|---|---|---|---|---|
| Enterococcus | 25% | 42%* | 29% | 21% |
| K.pneumoniae | 25%* | 7% | 12% | 8% |
| Gram neg rods | 39%* | 25% | 24% | 21% |
| Anaerobes | 26%* | 34% | 0 | 39% |

There were no significant differences in cure rate by prophylaxis group. However, cefazolin and other groups had a higher rate of wound infection
**CONCLUSIONS:** 1-3 doses of first generation β lactam antibiotics alter genital tract microflora. The use of 1-3 doses of ampicillin for group B streptococcus prophylaxis may have similar consequences. However, the clinical significance of changes in vaginal flora are poorly elucidated.

**PHAGE INFECTION IN VAGINAL LACTOBACILLI: AN *IN VITRO* MODEL.** SM Mou, MD; SI Pavlova, PhD; AO Kilic, PhD; and L Tao, PhD. University of Missouri-Kansas City, Kansas City, MO 64108

***Objectives:*** Studies documenting the harmful effects of phage infection in dairy lactobacilli are common; however, few studies have been conducted to determine if phage infections can have similar harmful effects on vaginal lactobacilli. The objectives of this study were to isolate phages from vaginal lactobacilli and establish an *in vitro* model to show that phages released from one woman's vaginal strains can infect lactobacilli from other women.

***Study Design:*** Vaginal samples were obtained from 39 reproductive-aged women (1 Native American, 5 Asians, 6 Blacks, and 27 Caucasians). The selective Rogosa agar was used to isolate lactobacilli, from which phages were induced by mitomycin C. Phage virulence was analyzed by cross-infecting these phages against a range of vaginal lactobacilli.

***Results:*** Of 19 samples from women with vaginal infections, 12 did not have lactobacilli. From the remaining 7 infection samples and the 20 samples from healthy women, 40 *Lactobacillus* stains were isolated, from which 6 *Lactobacillus* species were identified and 7 phages were isolated. One phage, ϕkc039, was characterized as follows: plaque morphology, small and clear, 1-2 mm in diameter; burst size, 300/cell; spontaneous induction rate, $10^{-6}$/cell; DNA, double stranded and linear, 41 kb; and shape, a hexagonal head and a non-contractile tail. This phage attacked *in vitro* 8 out of 39 vaginal *Lactobacillus* strains from other women. One strain, ϕkc013, was completely lysed by ϕkc039.

***Conclusions:*** Bacteriophages were isolated from human vaginal lactobacilli for the first time, and an *in vitro* phage-infection model was established. Further studies are needed to determine if phage infection in vaginal lactobacilli occurs *in vivo* in humans and if it is clinically significant.

(Supported by the Concerned Parents for AIDS Research/AmFAR and the University of Missouri Research Board.)

SUSCEPTIBILITY TESTING OF VAGINAL YEAST ISOLATES USING A BROTH MICRODILUTION BIOASSAY
J Gunter M.D., D. Dozier B.sc., S. Faro M.D., Ph.d.
Objective: Determine the MIC's of fluconazole, miconazole, and amphotencin B for vaginal yeast isolates using Alamar blue colorimetric broth microdilution.
Study Design: The test organisms consisted of 19 clinical isolates and 3 corresponding ATCC strains. The organisms (*C. albicans*, *T. glabrata*, and *C. tropicalis*) were isolated from the posterior vaginal fomix on Sabouraud dextrose agar, identified by standard methods and subcultured twice to ensure purity. The broth microdilution was performed according to the NCCLS standard guidelines with modification for the Alamar colorimetric method[1]. Stock solutions of antifungals were prepared in RPMI-1640. MIC's were read at 24 and 48 hours. The MIC was the lowest concentration of antifungal solution showing inhibition of growth. Sterility and growth controls were performed.
Results:

| Species | Antifungal agent | MIC (mcg/ml-range) 24 hrs | MIC (mcg/ml)-range 48 hrs |
|---|---|---|---|
| **C. albicans** | fluconazole | 0.25-1.0 | 0.25-2.0 |
| | amphotericin B | 0.625-0.25 | 0.25-0.50 |
| | miconazole | <0.05 | <0.50-0.098 |
| **T. glabrata** | fluconazole | 1.0-16.0 | 2.0-64.0 |
| | amphotericin B | <0.0313-0.625 | 0.125-0.50 |
| | miconazole | <0.050 | 0.195-12.5 |
| **C. tropicalis** | fluconazole | 1.0-2.0 | 4 0-16.0 |
| | amphotericin B | 0 125 | 0.50 |
| | miconazole | <0.050 | 0.391 |

Conclusions: Using this bioassay the MIC's for fluconazole, amphotencin B and miconazole were determined and found to be within the published NCCLS reference ranges. One *T. glabrata* wild type was resistant to fluconazole and miconazole but not to amphotericin B. We are currently increasing the number of isolates tested and correlating these in vitro results to clinical outcome.

[1] Tiballi RN, et al. Use of a Colorimetric System for Yeast Susceptibility Testing *J. Clin. Microbiol.* 33:915-917

**PROPHYLACTIC ANTIBIOTICS ARE NOT NECESSARY FOR MANUAL EXTRACTION OF PLACENTA FOLLOWING VAGINAL DELIVERY.**

M. McNamara,D.O., S. Hall, M.D., B. Gonik, M.D. Dept. Ob/Gyn, Wayne State Univ. Sch. of Med, Detroit, MI.

**OBJECTIVE:** Retained placenta is a common intrapartum complication following vaginal delivery which requires manual extraction (ME). Because of concerns related to bacterial contamination and subsequent infection, many clinicians administer antibiotic prophylaxis with this procedure. There are no data to support the need for antibiotic prophylaxis with ME.
**STUDY DESIGN:** This retrospective study examined 190 patients who underwent ME following vaginal delivery. Seventy one patients received prophylactic antibiotics and 119 patients did not. Indications for ME were prolonged third stage (123), retained products (16), bleeding (14), avulsed cord (14), and other (23). None of these patients received intrapartum antibiotics or were suspected of infection during their labor. Indications for ME, risk factors for infection and concomitant curettage were similar for both study groups. The decision to administer antibiotic prophylaxis was based on individual clinician preference. If antibiotics were given, they were administered from the time of ME up to 48 hours post procedure. Patients receiving antibiotic prophylaxis were compared to those not receiving prophylaxis for post partum infectious morbidity.
**RESULTS:** Three patients in the prophylaxis group developed post partum endometritis (4.2%). Of these three patients, one also had uterine curettage. One subject was admitted on post partum day 10 for suspected endometritis. All three had 24 hours of prophylactic antibiotics. No patient in the group not receiving antibiotics developed post partum endometritis.
**CONCLUSION:** These data suggest that ME following vaginal delivery does not place the patient at an increased risk for post partum endometritis. Therefore, prophylactic antibiotics are not necessary.

OVER-THE-COUNTER (OTC) AND ALTERNATIVE MEDICINES (AM) IN THE TREATMENT OF CHRONIC VAGINAL SYMPTOMS.

Paul Nyirjesy, MD, M. Velma Weitz, MSN; M.H. Terry Grody, MD; Bennett Lorber, MD. Temple University School of Medicine, Philadelphia, PA.

***Objective.*** To investigate the use of OTCs and AMs in patients with chronic vaginal symptoms.
***Study Design.*** A prospective cohort study of 109 patients referred by their gynecologists for evaluation of chronic vaginal symptoms. Patients were interviewed about their use of OTCs and AMs over the preceding year, the amount of money spent on each, and whether their physicians had been informed of these treatments.
***Results.*** The mean age was 35 ± 9.2 years; 50.9% had finished college. Symptom duration was 3.4 ± 3.5 years. 79 (72.5%) patients had self-treated with OTCs such as miconazole (75.6% of OTC users), clotrimazole (38.5%), or Betadine (14.1%). The average estimated expenditure for OTCs was $88 ± $139. 42 (38.5%) had used AMs, most frequently acidophilus pills orally (57.1%) or vaginally (11.9%), yogurt orally (21.4%) or vaginally (19.0%), garlic pills (9.6%) and acupuncture (4.8%). Estimated expenditure for AMs was $171 ± $284. Fewer physicians (79%) were aware of the use of AMs than of OTCs (96%) ($p<0.01$).
Although all patients had received at least one prescribed course of antimycotics for their conditions, the most common diagnoses at initial presentation were vulvovaginal candidiasis (VVC) in 28 (26.7%), vulvar vestibulitis in 19 (18.1%), irritant dermatitis in 18 (17%), and bacterial vaginosis in 12 (11.4%). Women who actually had VVC were more likely to have used AMs (RR 1.8, 95%CI 1.12-2.77) than other patients.
***Conclusions.*** Women with chronic vaginal symptoms often use OTCs and AMs which significantly add to health care costs and are unlikely to be of benefit.

**A RETROSPECTIVE ANALYSIS OF SEROLOGIC RESPONSE TO THE TREATMENT OF SYPHILIS DURING PREGNANCY.**

Henry L. Galan, MD, Juan Montalvo, MD, John Deaver, MD. Dept of Ob/Gyn, U.T. Medical School, Houston, TX..

**OBJECTIVE:** Treatment failures for syphilis during pregnancy continue to account for a high incidence of congenital syphilis. The purpose of this study was to assess the effect of several variables on the serologic response of syphilis following treatment in pregnancy.
**STUDY DESIGN:** A five-year chart review identified 95 patients coded with syphilis at Hermann Hospital. Inclusion criteria: 1) serologically confirmed syphilis infection index pregnancy, 2) treatment during the index pregnancy, and 3) completion of CDC recommended treatment during the index pregnancy. Forty-nine of 95 patients met the inclusion criteria. Titer responses were evaluated by assessing individual's follow-up titers to the pre-treatment titer as a single observation (dependent variable). Observations were compared in dichotomous fashion by classifying the response as either positive (≥4-fold decline) or negative (<4-fold decline). Independent variables were also dichotomized and included: 1) pregestational syphilis treated prior to the index pregnancy, 2) gestational age, 3) titer level, 4) unknown duration, 5) positive response at 1 month, 6) positive response at 2 months, 7) positive response at >3 months, 8) parity, 9) race. Analysis performed using Chi Square and Fisher's Exact tests with $p<0.05$ taken as significant.
**RESULTS:** A positive response was significantly more likely if there was no prior history of syphilis or if there was a high initial RPR titer (>32). Only 33/54 (61%) observations at three months or greater had a positive response.
**CONCLUSION:** Our study suggests that an absence of a history of syphilis and an initial high RPR titer are predictive of a positive response following recommended treatment. We speculate that we may be under-treating our pregnant patients infected with syphilis. Further studies are needed to evaluate the normal serologic response following syphilis treatment in pregnancy.

